# Estimation of unreported SARS-CoV-2 cases in Italy using a Susceptible-Exposed-Infectious-Recovered-Dead model

**DOI:** 10.7189/jogh.10.021105

**Published:** 2020-12

**Authors:** Andrea Maugeri, Martina Barchitta, Sebastiano Battiato, Antonella Agodi

**Affiliations:** 1 Department of Medical and Surgical Sciences and Advanced Technologies “GF Ingrassia”, University of Catania, Catania, Italy; 2 Department of Mathematics and Computer Science, University of Catania, Catania, Italy; 3 Azienda Ospedaliero Universitaria Policlinico “G. Rodolico-San Marco”, Catania, Italy

## Abstract

**Background:**

An important epidemiological characteristic that might modulate the pandemic potential of severe acute respiratory syndrome coronavirus 2 (SARS-CoV-2) is the proportion of undocumented cases.

**Methods:**

Here, we employed a Susceptible-Exposed-Infectious-Recovered-Dead (SEIRD) model to estimate the proportion of unreported SARS-CoV-2 cases in Italy from the reported number of deaths prior to the adoption of national control measures.

**Results:**

We estimated 115 894 infectious individuals (95% confidence interval (CI) = 95 318-140 455) and a total of 144 116 cases (95% CI = 119 030-173 959) on 20 March, 2020. These estimates resulted in 67.3% (95% CI = 60.3%-73.0%) unreported infectious individuals and in 67.4% (95% CI = 60.5%-73.0%) total cases. As such, given the substantial volume of undocumented cases, the case fatality risk would drop from an apparent 8.6% to an estimated 2.6% (95% CI = 2.2%-2.9%).

**Conclusions:**

Our findings partially explain the case fatality risk observed in Italy with a high proportion of unreported SARS-CoV-2 cases. Moreover, we underline that the fraction of undocumented infectious individuals is a critical epidemiological characteristic that needs to be taken into for a better understanding of the SARS-CoV-2 epidemic.

The epidemic of severe acute respiratory syndrome coronavirus 2 (SARS-CoV-2) emerged in Wuhan (Hubei province, China) in December 2019. At the end of February 2020, two distinct outbreaks occurred in two small Italian areas within the Lombardy and Veneto regions [1]. The epidemic, since then, spread to all the Italian regions, causing 10 149 cases and 631 deaths as of 10 March, 2020 [2]. On that day, the Italian government has promptly reacted by adopting a first set of control measures to the whole country (ie, travel restrictions, quarantine and contact precautions) [3,4], which were further tightened on 23 March (ie, restrictions of non-essential industrial productions and social interactions) [3,4].

Although efforts to contain the virus are still ongoing, there are many uncertainties regarding pathogen transmissibility and virulence that make it difficult to estimate the effectiveness of these strategies. An important epidemiological characteristic that might modulate the pandemic potential of SARS-CoV-2 is the proportion of undocumented cases, namely patients that often experience mild or no symptoms and hence. Indeed, undocumented cases could expose a far greater portion of the population to virus than would otherwise occur [5]. In the early phase of the epidemic, the Italian Ministry of Health recommended an extensive testing of all contacts of infectious patients. However, it has been later adopted a more stringent testing strategy of only patients who were suspected to be infected by SARS-CoV-2 and required hospitalization [5]. This strategy resulted in a high fraction of undocumented cases and an apparent increase in the case-fatality risk [5], that is the proportion of individuals diagnosed with SARS-CoV-2 who died.

In the last months, several epidemic models have been often employed to infer epidemiological characteristics or to evaluate the effectiveness of strategies against SARS-CoV-2 epidemic [6-13]. In Italy, some attempts to model the epidemic spread have first raised concern regarding the national health system’s capacity to address to the needs of patients [7,14]. Afterwards, further research concentrated efforts to estimate the effects of progressive restrictions [15]. In this scenario, we have previously proposed a Susceptible-Exposed-Infectious-Recovered-Dead (SEIRD) model to back-calculate the proportion of unreported SARS-CoV-2 cases from the reported number of deaths. Our hypothesis, in fact, is that the number of deaths is less likely to be affected by ascertainment biases than other data. We have already employed this model to estimate the proportion of unreported cases in China prior to the lockdown of Wuhan and Hubei province [16]. Specifically, our estimate of unreported SARS-CoV-2 infections – approximately 90% of total estimated infections – was in line with those proposed by other studies on Chinese data [8,11]. Here, we used this model to estimate the proportion of unreported SARS-CoV-2 cases in Italy before strategies against the epidemic have been effective. Given that control measures have been adopted on 10 March and considering a lag of at least ten days between the adoption of restrictions and their impact on death trend, we modelled the SARS-CoV-2 spread until 20 March, 2020.

## METHODS

### Formulating the SEIRD model

**Figure 1:**
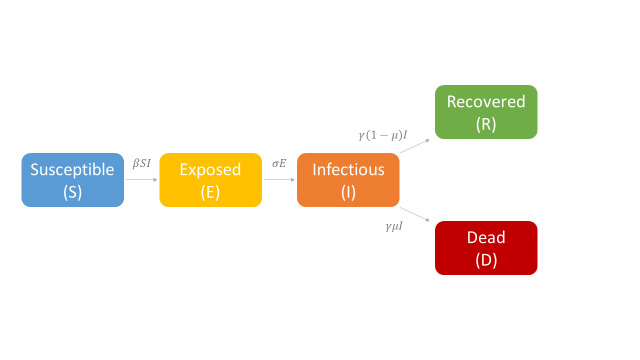
Illustration of the Susceptible-Exposed-Infectious-Recovered-Dead structure and equations employed to model the SARS-CoV-2 epidemic in Italy. β, σ; γ, and μ were transmission rate, infection rate, removing rate, and the probability of dying among infectious individuals, respectively.

Several models have been developed to describe the SARS-CoV-2 spread in specific countries or at global scale [6-11] but actually no consensus exists on the different compartments that should be considered. Here, we proposed and employed a model that was adapted from the standard susceptible-exposed-infectious-removed (SEIR) structure but distinguishing the removed state into recovered cases and deaths. By introducing the compartment of deaths, we accounted for a peculiar epidemiological state that was less affected by differences in testing strategies and hence less prone to ascertainment biases. The structure of our SEIRD model is depicted in **Figure 1****.**

Thus, the core of our model included the following compartments: Susceptible (*S*), Exposed (*E*), Infectious (*I*), Recovered (*R*), and Dead (*D*) individuals. In the model, susceptible individuals became exposed (ie, infected but not yet be infectious) to the viral agent upon contact with infectious cases. Infectious individuals (*I*) left the I compartment when they recovered from infection or died. Transmission dynamics are given by the following ordinary differential equations:


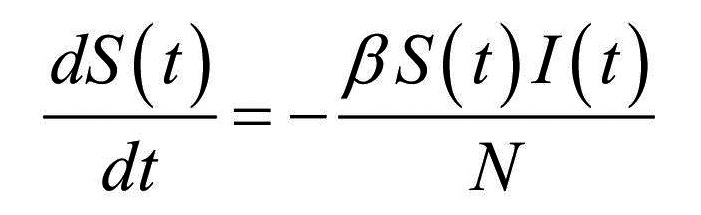
(1)


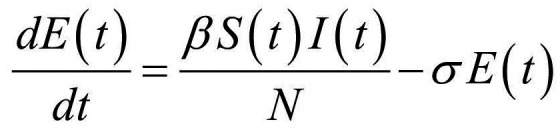
(2)


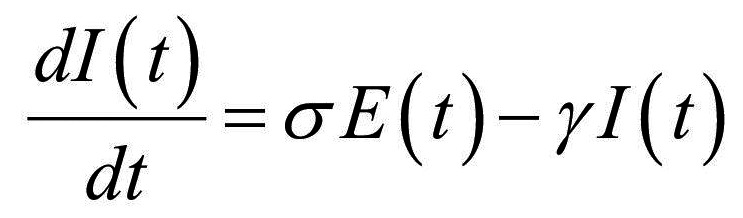
(3)


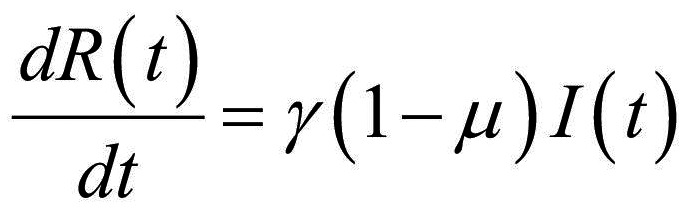
(4)


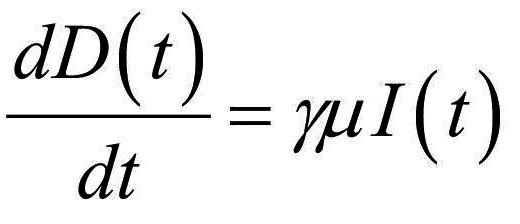
(5)

where:

- *N* was the total population, given by the sum of individuals in each compartment;
- *S(t)*, *E(t)*, *I(t)*, *R(t)*, and *D(t)* were the numbers of individuals in each compartment at time *(t)*;
- *β* was the transmission rate;
- *σ* was the infection rate and was assumed to be the inverse of the latency period (ie, the period from infection to the onset of symptoms);
- *γ* was the removing rate and was assumed to be the inverse of the period between the onset of symptoms and recovering/death;
- *μ* was the probability of dying among infectious individuals.

Specifically, equations (1) and (2) regulated the flow of individuals from *S* to *E* state according to the number of *S* and *I* individuals at each time *(t)*, the transmission rate β, and the total population *N*. Notably, *S* individuals could become *E* after contact with I individuals. Equation (3) regulated the flow of patients from *E* to *I* state according to the number of *E* individuals at each time *(t)*, and the infection rate. Equations (4) and (5) regulated the flow of patients from *I* to *R* or *D* states according to the number of *I* individuals at each time *(t)*, the removing rate and the probabilities of dying or surviving among *I* individuals.

### Fitting the SEIRD model to Italian data

In the current study, we modelled the SARS-CoV-2 epidemic in Italy from 25 January to 20 March, 2020. Our choice of the starting date corresponded to one mean latency period (ie, approximated to 5 days according to Li and colleagues [17]) before the first cases in Italy were announced on 29 January, 2020. The ending date was chosen considering a lag of ten days between the adoption of national control measures on 10 March 2020, and their impact on death trend. In our model, N was assumed to be the Italian population (60 million), R and D were initially set to 0, and the initial assumed number of infective individuals was set to 1. We assumed σ as 1/5.2 days according to Li and colleagues [17], while γ was set to 1/12 days based on previous estimates of infectious and hospitalization periods in China and Italy [9,18,19]. The initial ranges of the unknown model parameters were 0.1≤ β ≤1 and 0.001≤ μ ≤0.200, respectively.

Next, we fitted our model to the daily number of deaths from 24 February to 20 March, 2020, as reported by the Italy’s Civil Protection and archived on GitHub [2]. To estimate the best-fitting parameters with their 95% confidence interval (95% CI), we applied a least squares optimization using an evolutionary algorithm and simulations (n = 1000) on randomly generated samples from the distribution function of reported deaths. Specifically, the daily number of deaths followed a third-degree polynomial with R^2^ = 0.986. The algorithm was based on a population size = 1×10^5^, convergence = 1×10^−6^, and mutation rate = 5×10^−2^.

### Evaluation of unreported events and sensitivity analysis

Using the best-fitting parameters, we estimated the number of infections and total cases from 24 February to 20 March 2020. The proportions of unreported events were obtained by subtracting the reported numbers from those estimated. The basic reproductive number (R_0_) was calculated using the best-fitting parameters, as previously described [20]. We also did a sensitivity analysis to evaluate the impact of varying some parameters that might affect transmission dynamics and estimation of unreported cases and infections. Specifically, we maintained all the initial conditions of the baseline scenario but assuming different infectious period: respectively, half less (6 days) or half more (18 days) than that assumed in the baseline scenario. We also performed a sensitivity analysis by increasing the initial number of infectious individuals to 10.

### Ethics

This study used publicly available information with no personal identifiers. Therefore, informed consent and ethical approval were waived.

## RESULTS

### Description of reported events

**Figure 2:**
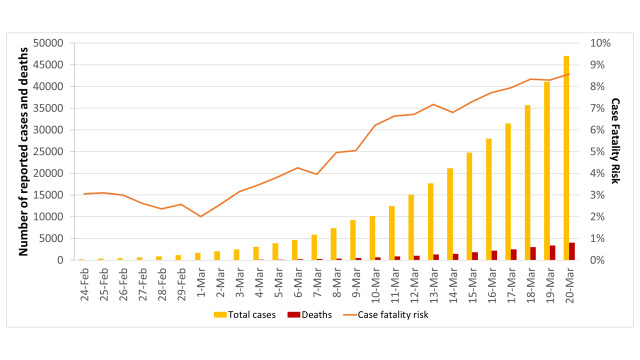
Number of SARS-CoV-2 cases and related deaths in Italy reported by the Italy’s Civil Protection from 24 February to 20 March 2020. The bars represent cumulative SARS-CoV-2 cases and deaths; the orange line represents the case fatality risk.

The daily numbers of cumulative SARS-CoV-2 cases and related deaths - reported by the Italy’s Civil Protection from 24 February to 20 March 2020 - are shown in **Figure 2**. Accordingly, we observed that the case fatality risk increased from 3.1% on February 24 to 8.6% on 20 March 2020. In order to explain this apparent increase in the case fatality risk, we hypothesized that a significant number of cases with mild or no symptoms has not been reported, thereby affecting the crude estimate.

### Model fitting and unreported SARS-CoV-2 cases

**Figure 3:**
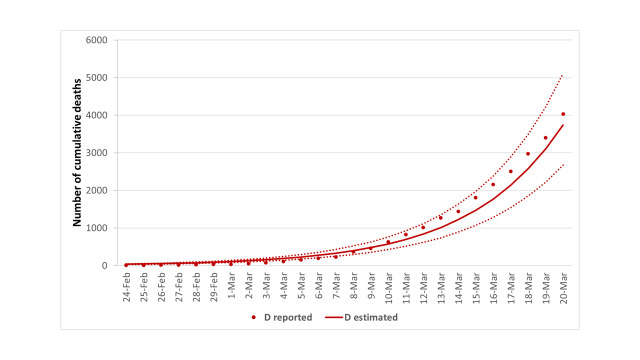
Fitting the SEIRD model to the cumulative deaths, reported by the Italy’s Civil Protection from 24 February to 20 March 2020. The dots represent the reported data while the lines represent the estimates with their 95% confidence intervals. D – deaths.

Thus, we first fitted our model to the reported number of cumulative deaths, which was certainly less prone to ascertainment biases. **Figure 3** suggested an overall good fit between estimated and reported deaths from 24 February to 20 March 2020 (Correlation Coefficient R^2^ = 0.991). Using the best-fitting parameters summarized in **Table 1**, our estimate of R_0_ was 4.0 (95% CI = 3.8-4.3) with an epidemic doubling time of 3.7 days (95% CI = 3.6-3.8).

**Table 1:** Assumed and estimated parameters of the Susceptible-Exposed-Infectious-Recovered-Dead model

SEIRD model	*S*	*E*	*I*	*R*	*D*	β*	σ	γ	μ†
Baseline Scenario	59 999 999	0	1	0	0	0.32 (95% CI = 0.30-0.34)	0.19‡	0.08§	0.13 (95% CI = 0.11-0.15)
Sensitivity analysis, Scenario 1	59 999 999	0	1	0	0	0.26 (95% CI = 0.25-0.28)	0.19‡	0.17‖	0.16 (95% CI 0.14-0.18)
Sensitivity analysis, Scenario 2	59 999 999	0	1	0	0	0.50 (95% CI = 0.49-0.51)	0.19‡	0.06¶	0.15 (95% CI = 0.13-0.16)
Sensitivity analysis, Scenario 3g	59 999 999	0	10	0	0	0.30 (95% CI = 0.28-0.32)	0.19‡	0.08**	0.11 (95% CI = 0.09-0.13)

S – Susceptible, E – exposed, I – infectious, R – recovered, D – deaths, CI – confidence interval

* Estimated through the model with a potential range 0.1≤ β ≤1.0.

†Estimated through the model with a potential range 0.01≤ μ ≤0.20.

‡Assumed to be 1/5.2 d according to Li and colleagues [8].

§Assumed to be 1/12 d according to previous estimates [9,18,19].

‖Assuming that infectious period was half less than the baseline scenario.

¶Assuming that infectious period was half more than the baseline scenario.

**Assuming that initial number of infectious individuals was 10.

**Figure 4:**
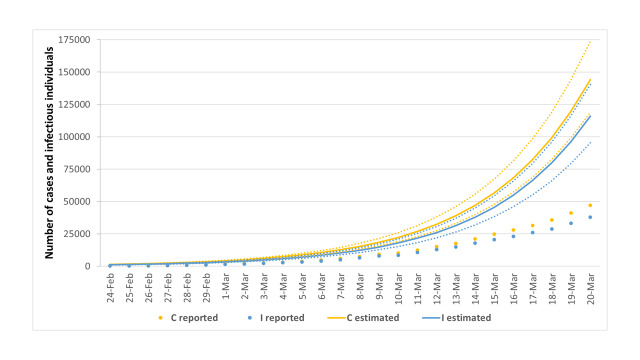
Estimation of the number of infectious individuals and total SARS-CoV-2 cases in Italy from 24 February to 20 March 2020. The dots represent data reported by the Italy’s Civil Protection, while the lines represent the estimates with their 95% confidence intervals. I – infectious individuals, C – cases.

**Figure 4** depicts the comparison between reported and estimated number of infectious individuals and total SARS-CoV-2 cases. Specifically, we estimated 115 894 infectious individuals (95% CI = 95 318-140 455) and a total of 144 116 cases (95% CI = 119 030-173 959) on March 20.

**Figure 5:**
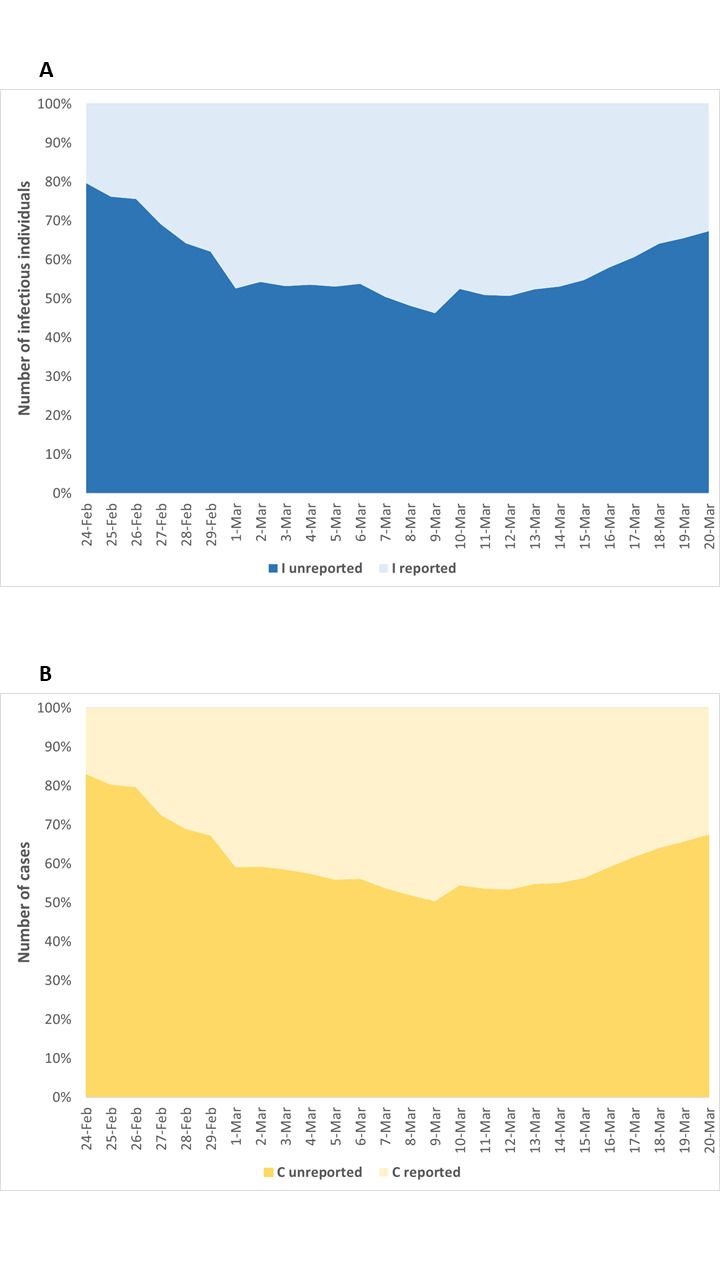
Proportion of unreported infectious individuals (**A**) and total SARS-CoV-2 cases (**B**) in Italy from 24 February to 20 March 2020. I – infectious individuals, C – cases.

In line with these estimates, the proportions of unreported infectious individuals and total SARS-CoV-2 cases on March 20 were 67.3% (95% CI = 60.3%-73.0%) and 67.4% (95% CI = 60.5%-73.0%), respectively (**Figure 5**). As such, given the substantial volume of undocumented cases, the estimated case fatality risk would drop from 8.6% to 2.6% (95% CI = 2.2%-2.9%).

### Sensitivity analysis

The sensitivity analyses confirmed that our model was robust, so that changes in the infectious period or in the initial number of infectious individuals led to the readjustment of best-fitting parameters (**Table 1**). Despite a different infectious period, however, the proportion of unreported cases remained almost unchanged, ranging from 66.4% (95% CI = 62.2%-70.0%) in the scenario with γ = 1/6 days to 72.5% (95% CI = 66.5%-77.3%) in the scenario with γ = 1/18 days. Similarly, the proportion of unreported cases was 68.5% (95% CI = 61.3%-73.8%) in the scenario with 10 infectious individuals on the starting date.

## DISCUSSION

Compared with other countries, Italy currently has a very high case fatality risk due to SARS-CoV-2 infection, which also increased as the epidemic spread. However, in a previous viewpoint, Onder and colleagues suggested that different testing strategies between and within countries might partially explain the apparent increase in the case fatality risk observed in Italy [5]. Moreover, other studies reported that substantial undocumented infections could facilitate the rapid dissemination of SARS-CoV-2 [8]. In the current study, we employed a SEIRD model to estimate the proportion of unreported SARS-CoV-2 cases in Italy from 24 February to 20 March, 2020. Our choice of this ending date was motivated by assuming a delay of 1-2 weeks between the adoption of national control measures on 10 March 2020, and their impact on the epidemic curve. We previously used the same model to estimate the proportion of unreported SARS-CoV-2 cases in China prior to the lockdown of Wuhan and Hubei province [16]. Interestingly, our estimates on the Chinese epidemic were almost aligned with those obtained by applying other models [8,11]. In this study, we estimated approximately 144 000 SARS-CoV-2 cases in Italy on March 20, which resulted in 67.4% unreported cases. The novelty of our approach relied on using a compartmental model, which distinguished the removed state into recovered and dead individuals. Indeed, to our knowledge, our study was the first that applied a SEIRD model to estimate the epidemic curve in Italy, working on observed deaths. A similar approach was used by the Imperial College COVID-19 Response Team that is currently investigating the SARS-CoV-2 epidemic by back-calculating from deaths observed over time [21]. Indeed, data on the cumulative number of deaths were certainly less prone to ascertainment biases than those on infections.(21) Consistency of our model was also corroborated the an estimated R_0_ of 4, which was in line with previous estimates indicating a high capacity for sustained transmission at the beginning of the epidemic in Italy and other countries [6,8,10,21,22].

Given the substantial volume of undocumented cases, the estimated case fatality risk would drop from 8.6 to 2.6%. Although this estimate was more aligned to those reported in other countries, it still remained higher. The residual difference could be partially attributed to the overall older age distribution in Italy if compared with other countries and/or to different definitions of SARS-CoV-2 related deaths [5].

We recognized that our findings were based on an epidemic model and that some limitations should be considered when interpreting our results. Although we hypothesized that data on deaths were less prone to underreporting than those on other events (eg, cases, infections, recovered patients), it was not exempt from ascertainment biases. Specifically, no consensus existed on a clear definition of SARS-CoV-2 related death [5], and hence it might be possible that some deaths were caused by preexisting diseases or conditions rather than SARS-CoV-2 infection. However, our model did not account for a causal relationship between SARS-CoV-2 infection and deaths, but only on the probability of death among infectious individuals. Thus, different definitions of SARS-CoV-2 related death might affect this probability but not the model itself. Another parameter regulating the transition from the infectious to the removed state was the removing rate, which was assumed to be the inverse of the infectious period. Since no consensus existed on this parameter, we did a sensitivity analysis using alternative removing rates. However, our model was robust and insensitive to these changes. Finally, we recognized that our model relied on assumptions and several parameters that had to be fixed. Although we provided reasonable rationale and appropriate references motivating our choices, we cannot completely exclude some degree of uncertainty of our estimates.

Our findings raise the need for transparent and accurate reporting of testing strategies that might improve our understanding of global SARS-CoV-2 epidemic. Indeed, the fraction of undocumented but infectious cases is a critical epidemiological characteristic that needs to be taken into account in the development of effective strategies to drastically reduce within-population contact rates.
